# Supplementary material for: Analytical solution for a hybrid Logistic‐Monod cell growth model in batch and continuous stirred tank reactor culture
Source: Biotechnol Bioeng. 2019 Dec 2;117(3):873–8. doi: 10.1002/bit.27230 (PMC7027892; doi:10.1002/bit.27230)
Supplement: Supplementary file 1 — Supplementary information [file BIT-117-873-s001.docx]

***Supplementary files***

**Communications to the Editor**

**Analytical solution for a hybrid Logistic-Monod cell growth model in batch and CSTR culture**

**Running title: Analytical solution for hybrid Logistic-Monod model**

Peng Xu*

Department of Chemical, Biochemical and Environmental Engineering, University of Maryland Baltimore County, Baltimore, MD 21250

* Corresponding author, Tel: +1(410)-455-2474; fax: +1(410)-455-1049. E-mail address: pengxu@umbc.edu (PX)

## **1. Matlab code to solve the Monod growth model in a batch fermentation**

>> syms mu_m K_S X(t) X0 S0 Y_xs S(t)

eqn5 = diff(X,t) == mu_m*(S0-(X-X0)/Y_xs)*X/(K_S+ S0-(X-X0)/Y_xs)

cond1 = X(0) == X0;

solX =dsolve(eqn5,cond1);

eqn5(t) =

diff(X(t), t) == (mu_m*X(t)*(S0 + (X0 - X(t))/Y_xs))/(K_S + S0 + (X0 - X(t))/Y_xs)

>> latex(eqn5)

ans =

$$\frac{\partial}{\partial t}X\left( t \right)=\frac{\mu_{m} X\left( t \right) \left( S_{0}+\frac{X_{0}-X\left( t \right)}{Y_{\text{xs}}} \right)}{K_{S}+S_{0}+\frac{X_{0}-X\left( t \right)}{Y_{\text{xs}}}}$$

solX =

solve(log(X) - log(X0) - mu_m*t - (2*K_S*Y_xs*atanh((X0 - 2*X + S0*Y_xs)/(X0 + S0*Y_xs)))/(X0 + S0*Y_xs) - (2*K_S*Y_xs*atanh((X0 - S0*Y_xs)/(X0 + S0*Y_xs)))/(X0 + S0*Y_xs), X)

>> latex(solX)

ans =

$$\text{solve}\left( \ln\left( X \right)-\frac{2 K_{S} Y_{\text{xs}} \text{atanh}\left( \frac{X_{0}-2 X+S_{0} Y_{\text{xs}}}{X_{0}+S_{0} Y_{\text{xs}}} \right)}{X_{0}+S_{0} Y_{\text{xs}}}=\ln\left( X_{0} \right)+\mu_{m} t+\frac{2 K_{S} Y_{\text{xs}} \text{atanh}\left( \frac{X_{0}-S_{0} Y_{\text{xs}}}{X_{0}+S_{0} Y_{\text{xs}}} \right)}{X_{0}+S_{0} Y_{\text{xs}}},X \right)$$

>> syms mu_m K_S X X0 S0 Y_xs S

>> eqn1 = log(X/X0) == mu_m*t + K_S*Y_xs/(X0+S0*Y_xs)*log((X0/(S0*Y_xs))*(X0-X+S0*Y_xs)/X);

>> latex(eqn1)

$$\ln\left( \frac{X}{X_{0}} \right)=\mu_{m} t+\frac{K_{S} Y_{\text{xs}}}{X_{0}+S_{0} Y_{\text{xs}}}\ln\left( \frac{X_{0} \left( X_{0}-X+S_{0} Y_{\text{xs}} \right)}{S_{0} X Y_{\text{xs}}} \right)$$

>> solS = S0-(solX-X0)/Y_xs

solS =

{S0 + (X0 - z)/Y_xs | z in solve(log(X) - (2*K_S*Y_xs*atanh((- 2*X + X0 + S0*Y_xs)/(X0 + S0*Y_xs)))/(X0 + S0*Y_xs) == log(X0) + mu_m*t + (2*K_S*Y_xs*atanh((X0 - S0*Y_xs)/(X0 + S0*Y_xs)))/(X0 + S0*Y_xs), X)}

>> latex(solS)

ans =

$$\left\{ S_{0}+\frac{X_{0}-z}{Y_{\text{xs}}} | z\in\text{solve}\left( \ln\left( X \right)-\frac{2 K_{S} Y_{\text{xs}} \text{atanh}\left( \frac{X_{0}-2 X+S_{0} Y_{\text{xs}}}{X_{0}+S_{0} Y_{\text{xs}}} \right)}{X_{0}+S_{0} Y_{\text{xs}}}=\ln\left( X_{0} \right)+\mu_{m} t+\frac{2 K_{S} Y_{\text{xs}} \text{atanh}\left( \frac{X_{0}-S_{0} Y_{\text{xs}}}{X_{0}+S_{0} Y_{\text{xs}}} \right)}{X_{0}+S_{0} Y_{\text{xs}}},X \right) \right\}$$

## **2. Matlab code to solve the Logistic growth model in a batch fermentation**

>> syms mu_m X_m X(t) X0 S0 Y_xs S(t)

>> eqn1 = diff(X,t) == mu_m*X*(1-X/X_m)

>> eqn2 = diff(S,t) == - mu_m*X*(1-X/X_m)/Y_xs;

>> cond1 = X(0) == X0;

>> cond2 = S(0) == S0;

>> eqns = [eqn1, eqn2]

>> conds =[cond1, cond2]

>> solLogistic =dsolve(eqns,conds);

>> eqn3 = X(t) == simplify(solLogistic.X);

>> eqn4 = S(t) == simplify(solLogistic.S);

>> latex(eqn1)

ans =

$$\frac{\partial}{\partial t}X\left( t \right)=-\mu_{m} X\left( t \right) \left( \frac{X\left( t \right)}{X_{m}}-1 \right)$$

>> latex(eqn2)

ans =

$$\frac{\partial}{\partial t}S\left( t \right)=\frac{\mu_{m} X\left( t \right) \left( \frac{X\left( t \right)}{X_{m}}-1 \right)}{Y_{\text{xs}}}$$

>> latex(eqn3)

ans =

$$X\left( t \right)=\frac{X_{0} X_{m} \text{e}^{\mu_{m} t}}{X_{m}-X_{0}+X_{0} \text{e}^{\mu_{m} t}}$$

>> latex(eqn4)

ans =

$$S\left( t \right)=\frac{X_{0} X_{m}-{X_{0}}^{2}+{X_{0}}^{2} \text{e}^{\mu_{m} t}-S_{0} X_{0} Y_{\text{xs}}+S_{0} X_{m} Y_{\text{xs}}-X_{0} X_{m} \text{e}^{\mu_{m} t}+S_{0} X_{0} Y_{\text{xs}} \text{e}^{\mu_{m} t}}{Y_{\text{xs}} \left( X_{m}-X_{0}+X_{0} \text{e}^{\mu_{m} t} \right)}$$

## **3. Matlab code to solve the Logistic-Monod Model for Batch culture**

>> syms mu_m K_S X_m X(t) X0 S0 Y_xs S(t)

>> eqn6 = diff(X,t) == mu_m*(S0-(X-X0)/Y_xs)*(1-X/X_m)*X/(K_S+ S0-(X-X0)/Y_xs)

>> cond1 = X(0) == X0;

>> solX =simplify(dsolve(eqn6,cond1));

>> latex(eqn6)

ans =

$$\frac{\partial}{\partial t}X\left( t \right)=-\frac{\mu_{m} X\left( t \right) \left( \frac{X\left( t \right)}{X_{m}}-1 \right) \left( S_{0}+\frac{X_{0}-X\left( t \right)}{Y_{\text{xs}}} \right)}{K_{S}+S_{0}+\frac{X_{0}-X\left( t \right)}{Y_{\text{xs}}}}$$

>> solX

solX =

log(X/X0)*(1/X_m + (K_S*Y_xs)/(X0*X_m + S0*X_m*Y_xs)) + log((X0 - X_m)/(X - X_m))*(1/X_m + (K_S*Y_xs)/(X0*X_m - X_m^2 + S0*X_m*Y_xs)) + (K_S*Y_xs*log((X - X0 - S0*Y_xs)/(-S0*Y_xs)))/(S0^2*Y_xs^2 + 2*S0*X0*Y_xs - X_m*S0*Y_xs + X0^2 - X_m*X0) - (mu_m*t)/X_m

>> latex(solX)

ans =

$$\ln\left( \frac{X}{X_{0}} \right) \left( \frac{1}{X_{m}}+\frac{K_{S} Y_{\text{xs}}}{X_{0} X_{m}+S_{0} X_{m} Y_{\text{xs}}} \right)+\ln\left( \frac{X_{0}-X_{m}}{X-X_{m}} \right) \left( \frac{1}{X_{m}}+\frac{K_{S} Y_{\text{xs}}}{X_{0} X_{m}-{X_{m}}^{2}+S_{0} X_{m} Y_{\text{xs}}} \right)+\frac{K_{S} Y_{\text{xs}} \ln\left( \frac{X-X_{0}-S_{0} Y_{\text{xs}}}{-S_{0} Y_{\text{xs}}} \right)}{{S_{0}}^{2} {Y_{\text{xs}}}^{2}+2 S_{0} X_{0} Y_{\text{xs}}-X_{m} S_{0} Y_{\text{xs}}+{X_{0}}^{2}-X_{m} X_{0}}=\frac{\mu_{m} t}{X_{m}}$$

$$\text{solve}\left( \frac{\ln\left( X \right) \left( X_{0}+K_{S} Y_{\text{xs}}+S_{0} Y_{\text{xs}} \right)}{X_{m} \left( X_{0}+S_{0} Y_{\text{xs}} \right)}-\frac{\ln\left( X-X_{m} \right) \left( X_{0}-X_{m}+K_{S} Y_{\text{xs}}+S_{0} Y_{\text{xs}} \right)}{X_{m} \left( X_{0}-X_{m}+S_{0} Y_{\text{xs}} \right)}+\frac{K_{S} Y_{\text{xs}} \ln\left( X-X_{0}-S_{0} Y_{\text{xs}} \right)}{\left( X_{0}+S_{0} Y_{\text{xs}} \right) \left( X_{0}-X_{m}+S_{0} Y_{\text{xs}} \right)}=\frac{\mu_{m} t}{X_{m}}-\frac{\ln\left( X_{0}-X_{m} \right) \left( X_{0}-X_{m}+K_{S} Y_{\text{xs}}+S_{0} Y_{\text{xs}} \right)}{X_{m} \left( X_{0}-X_{m}+S_{0} Y_{\text{xs}} \right)}+\frac{\ln\left( X_{0} \right) \left( X_{0}+K_{S} Y_{\text{xs}}+S_{0} Y_{\text{xs}} \right)}{X_{m} \left( X_{0}+S_{0} Y_{\text{xs}} \right)}+\frac{K_{S} Y_{\text{xs}} \ln\left( -S_{0} Y_{\text{xs}} \right)}{\left( X_{0}+S_{0} Y_{\text{xs}} \right) \left( X_{0}-X_{m}+S_{0} Y_{\text{xs}} \right)},X \right)$$

>> eqn7 = diff(S,t) == -mu_m*S*(1-(X0+Y_xs*(S0-S))/X_m)*(X0+Y_xs*(S0-S))/(K_S+ S)/Y_xs;

>> cond2 = S(0) == S0;

>> solS = dsolve(eqn7,cond2);

>> latex(eqn7)

ans =

$$\frac{\partial}{\partial t}S\left( t \right)=\frac{\mu_{m} S\left( t \right) \left( X_{0}+Y_{\text{xs}} \left( S_{0}-S\left( t \right) \right) \right) \left( \frac{X_{0}+Y_{\text{xs}} \left( S_{0}-S\left( t \right) \right)}{X_{m}}-1 \right)}{Y_{\text{xs}} \left( K_{S}+S\left( t \right) \right)}$$

solS =

(K_S*log(S/S0))/(S0^2*Y_xs^2 + 2*S0*X0*Y_xs - X_m*S0*Y_xs + X0^2 - X_m*X0) + (log((X0 - X_m)/(X0 - X_m - S*Y_xs + S0*Y_xs))*(X0 - X_m + Y_xs*(K_S + S0)))/(- X_m^2*Y_xs + S0*X_m*Y_xs^2 + X0*X_m*Y_xs) + (log((X0 - S*Y_xs + S0*Y_xs)/S0)*(X0 + Y_xs*(K_S + S0)))/(S0*X_m*Y_xs^2 + X0*X_m*Y_xs) - (mu_m*t)/(X_m*Y_xs)

>> latex(solS)

ans =

$$\frac{K_{S} \ln\left( \frac{S}{S_{0}} \right)}{{S_{0}}^{2} {Y_{\text{xs}}}^{2}+2 S_{0} X_{0} Y_{\text{xs}}-X_{m} S_{0} Y_{\text{xs}}+{X_{0}}^{2}-X_{m} X_{0}}+\frac{\ln\left( \frac{X_{0}-X_{m}}{X_{0}-X_{m}-S Y_{\text{xs}}+S_{0} Y_{\text{xs}}} \right) \left( X_{0}-X_{m}+Y_{\text{xs}} \left( K_{S}+S_{0} \right) \right)}{-{X_{m}}^{2} Y_{\text{xs}}+S_{0} X_{m} {Y_{\text{xs}}}^{2}+X_{0} X_{m} Y_{\text{xs}}}+\frac{\ln\left( \frac{X_{0}-S Y_{\text{xs}}+S_{0} Y_{\text{xs}}}{X_{0}} \right) \left( X_{0}+Y_{\text{xs}} \left( K_{S}+S_{0} \right) \right)}{S_{0} X_{m} {Y_{\text{xs}}}^{2}+X_{0} X_{m} Y_{\text{xs}}}=\frac{\mu_{m} t}{X_{m} Y_{\text{xs}}}$$

$$\frac{K_{S} \ln\left( \frac{S\left( t \right)}{S_{0}} \right)}{{S_{0}}^{2} {Y_{\text{xs}}}^{2}+2 S_{0} X_{0} Y_{\text{xs}}-X_{m} S_{0} Y_{\text{xs}}+{X_{0}}^{2}-X_{m} X_{0}}+\frac{\ln\left( \frac{X_{0}-X_{m}}{X_{0}-X_{m}+S_{0} Y_{\text{xs}}-Y_{\text{xs}} S\left( t \right)} \right) \left( X_{0}-X_{m}+Y_{\text{xs}} \left( K_{S}+S_{0} \right) \right)}{-{X_{m}}^{2} Y_{\text{xs}}+S_{0} X_{m} {Y_{\text{xs}}}^{2}+X_{0} X_{m} Y_{\text{xs}}}+\frac{\ln\left( \frac{X_{0}+S_{0} Y_{\text{xs}}-Y_{\text{xs}} S\left( t \right)}{S_{0}} \right) \left( X_{0}+Y_{\text{xs}} \left( K_{S}+S_{0} \right) \right)}{S_{0} X_{m} {Y_{\text{xs}}}^{2}+X_{0} X_{m} Y_{\text{xs}}}-\frac{\mu_{m} t}{X_{m} Y_{\text{xs}}}$$

$$\text{solve}\left( \frac{K_{S} \ln\left( S \right)}{{S_{0}}^{2} {Y_{\text{xs}}}^{2}+2 S_{0} X_{0} Y_{\text{xs}}-X_{m} S_{0} Y_{\text{xs}}+{X_{0}}^{2}-X_{m} X_{0}}-\frac{\ln\left( X_{0}-X_{m}-S Y_{\text{xs}}+S_{0} Y_{\text{xs}} \right) \left( X_{0}-X_{m}+Y_{\text{xs}} \left( K_{S}+S_{0} \right) \right)}{-{X_{m}}^{2} Y_{\text{xs}}+S_{0} X_{m} {Y_{\text{xs}}}^{2}+X_{0} X_{m} Y_{\text{xs}}}+\frac{\ln\left( X_{0}-S Y_{\text{xs}}+S_{0} Y_{\text{xs}} \right) \left( X_{0}+Y_{\text{xs}} \left( K_{S}+S_{0} \right) \right)}{S_{0} X_{m} {Y_{\text{xs}}}^{2}+X_{0} X_{m} Y_{\text{xs}}}=\frac{\ln\left( X_{0} \right) \left( X_{0}+Y_{\text{xs}} \left( K_{S}+S_{0} \right) \right)}{S_{0} X_{m} {Y_{\text{xs}}}^{2}+X_{0} X_{m} Y_{\text{xs}}}+\frac{K_{S} \ln\left( S_{0} \right)}{{S_{0}}^{2} {Y_{\text{xs}}}^{2}+2 S_{0} X_{0} Y_{\text{xs}}-X_{m} S_{0} Y_{\text{xs}}+{X_{0}}^{2}-X_{m} X_{0}}-\frac{\ln\left( X_{0}-X_{m} \right) \left( X_{0}-X_{m}+Y_{\text{xs}} \left( K_{S}+S_{0} \right) \right)}{-{X_{m}}^{2} Y_{\text{xs}}+S_{0} X_{m} {Y_{\text{xs}}}^{2}+X_{0} X_{m} Y_{\text{xs}}}+\frac{\mu_{m} t}{X_{m} Y_{\text{xs}}},S \right)$$

## **4. Matlab code to solve the hybrid Logistic-Monod cell growth model in CSTR**

>> syms S X D mu_max X_max S_F K_S Y_xs mu

>> eqn1 = mu == mu_max*(1-X/X_max)*S/(K_S+S)

>> eqn2 = mu*X-D*X == 0

>> eqn3 = -mu*X/Y_xs +D*(S_F-S) == 0;

>> eqns =[eqn1,eqn2, eqn3];

>> vars = [mu S X];

>> [solmu, solS, solX] = solve(eqns, vars)

>> latex(eqn1)

ans =

$$\mu=-\frac{S \mu_{\text{max}} \left( \frac{X}{X_{\text{max}}}-1 \right)}{K_{S}+S}$$

>> latex(eqn2)

ans =

$$X \mu-D X=0$$

>> latex(eqn3)

ans =

$$-D \left( S-S_{F} \right)-\frac{X \mu}{Y_{\text{xs}}}=0$$

>> latex(solS(2,1))

$S_{F}-\frac{\sqrt{D^{2} {X_{\text{max}}}^{2}+2 D S_{F} X_{\text{max}} Y_{\text{xs}} \mu_{\text{max}}-2 D {X_{\text{max}}}^{2} \mu_{\text{max}}+4 K_{S} D X_{\text{max}} Y_{\text{xs}} \mu_{\text{max}}+{S_{F}}^{2} {Y_{\text{xs}}}^{2} {\mu_{\text{max}}}^{2}-2 S_{F} X_{\text{max}} Y_{\text{xs}} {\mu_{\text{max}}}^{2}+{X_{\text{max}}}^{2} {\mu_{\text{max}}}^{2}}+X_{\text{max}} \mu_{\text{max}}-D X_{\text{max}}+S_{F} Y_{\text{xs}} \mu_{\text{max}}}{2 Y_{\text{xs}} \mu_{\text{max}}}$

>> latex(solS(3,1))

$S_{F}+\frac{\sqrt{D^{2} {X_{\text{max}}}^{2}+2 D S_{F} X_{\text{max}} Y_{\text{xs}} \mu_{\text{max}}-2 D {X_{\text{max}}}^{2} \mu_{\text{max}}+4 K_{S} D X_{\text{max}} Y_{\text{xs}} \mu_{\text{max}}+{S_{F}}^{2} {Y_{\text{xs}}}^{2} {\mu_{\text{max}}}^{2}-2 S_{F} X_{\text{max}} Y_{\text{xs}} {\mu_{\text{max}}}^{2}+{X_{\text{max}}}^{2} {\mu_{\text{max}}}^{2}}-X_{\text{max}} \mu_{\text{max}}+D X_{\text{max}}-S_{F} Y_{\text{xs}} \mu_{\text{max}}}{2 Y_{\text{xs}} \mu_{\text{max}}}$

$$S_{F}+\frac{\sqrt{D^{2} {X_{m}}^{2}+2 D S_{F} X_{m} Y_{\text{xs}} \mu_{m}-2 D {X_{m}}^{2} \mu_{m}+4 K_{S} D X_{m} Y_{\text{xs}} \mu_{m}+{S_{F}}^{2} {Y_{\text{xs}}}^{2} {\mu_{m}}^{2}-2 S_{F} X_{m} Y_{\text{xs}} {\mu_{m}}^{2}+{X_{m}}^{2} {\mu_{m}}^{2}}-X_{m} \mu_{m}+D X_{m}-S_{F} Y_{\text{xs}} \mu_{m}}{2 Y_{\text{xs}} \mu_{m}}$$

$$\frac{\sqrt{\left( \left( D-\mu_{m} \right)X_{m}+ S_{F} Y_{\text{xs}} \mu_{m} \right)^{2}+4 K_{S} D X_{m} Y_{\text{xs}} \mu_{m}}-X_{m} \mu_{m}+D X_{m}+S_{F} Y_{\text{xs}} \mu_{m}}{2 Y_{\text{xs}} \mu_{m}}$$

$$\frac{\sqrt{\left( X_{m} \left( D-\mu_{m} \right)+S_{F} Y_{\text{xs}} \mu_{m} \right)^{2}+4 D K_{S} X_{m} Y_{\text{xs}} \mu_{m}}-X_{m} \mu_{m}+D X_{m}+S_{F} Y_{\text{xs}} \mu_{m}}{2 Y_{\text{xs}} \mu_{m}}$$

>> latex(solX(2,1))

$\frac{\sqrt{D^{2} {X_{\text{max}}}^{2}+2 D S_{F} X_{\text{max}} Y_{\text{xs}} \mu_{\text{max}}-2 D {X_{\text{max}}}^{2} \mu_{\text{max}}+4 K_{S} D X_{\text{max}} Y_{\text{xs}} \mu_{\text{max}}+{S_{F}}^{2} {Y_{\text{xs}}}^{2} {\mu_{\text{max}}}^{2}-2 S_{F} X_{\text{max}} Y_{\text{xs}} {\mu_{\text{max}}}^{2}+{X_{\text{max}}}^{2} {\mu_{\text{max}}}^{2}}+X_{\text{max}} \mu_{\text{max}}-D X_{\text{max}}+S_{F} Y_{\text{xs}} \mu_{\text{max}}}{2 \mu_{\text{max}}}$

>> latex(solX(3,1))

$-\frac{\sqrt{D^{2} {X_{\text{max}}}^{2}+2 D S_{F} X_{\text{max}} Y_{\text{xs}} \mu_{\text{max}}-2 D {X_{\text{max}}}^{2} \mu_{\text{max}}+4 K_{S} D X_{\text{max}} Y_{\text{xs}} \mu_{\text{max}}+{S_{F}}^{2} {Y_{\text{xs}}}^{2} {\mu_{\text{max}}}^{2}-2 S_{F} X_{\text{max}} Y_{\text{xs}} {\mu_{\text{max}}}^{2}+{X_{\text{max}}}^{2} {\mu_{\text{max}}}^{2}}-X_{\text{max}} \mu_{\text{max}}+D X_{\text{max}}-S_{F} Y_{\text{xs}} \mu_{\text{max}}}{2 \mu_{\text{max}}}$

$$-\frac{\sqrt{\left( \left( D-\mu_{m} \right)X_{m}+ S_{F} Y_{\text{xs}} \mu_{m} \right)^{2}+4 K_{S} D X_{m} Y_{\text{xs}} \mu_{m}}-X_{m} \mu_{m}+D X_{m}-S_{F} Y_{\text{xs}} \mu_{m}}{2 \mu_{m}}$$

$$\frac{X_{m} \mu_{m}-\sqrt{\left( X_{m} \left( D-\mu_{m} \right)+S_{F} Y_{\text{xs}} \mu_{m} \right)^{2}+4 D K_{S} X_{m} Y_{\text{xs}} \mu_{m}}-D X_{m}+S_{F} Y_{\text{xs}} \mu_{m}}{2 \mu_{m}}$$

## **5. Matlab code to generate Figure 2**

syms K_S D S Y_xs mu_m X_m S_F X

S1 = K_S*D/(mu_m-D);

X1 = Y_xs*(S_F-K_S*D/(mu_m-D));

subplot(1,3,1)

fplot(subs(S1, [mu_m, X_m, S_F, K_S, Y_xs], [1.6, 10, 20, 1, 0.8]), [0 1.6] , 'LineWidth',2.0)

hold on

fplot(subs(X1, [mu_m, X_m, S_F, K_S, Y_xs], [1.6, 10, 20, 1, 0.8]), [0 1.6] , 'LineWidth',2.0)

fplot(subs(S1, [mu_m, X_m, S_F, K_S, Y_xs], [1.6, 10, 20, 1, 0.3]), [0 1.6] , 'LineWidth',2.0)

fplot(subs(X1, [mu_m, X_m, S_F, K_S, Y_xs], [1.6, 10, 20, 1, 0.3]), [0 1.6] , 'LineWidth',2.0)

>> xlabel('Dilution rate (1/h)')

ylabel('Cell and substrate (g/L)')

legend('Y_{xs}=0.8', 'Y_{xs}=0.8', 'Y_{xs}=0.3', 'Y_{xs}=0.3')

title('(a)');

ylim([0 16])

legend boxoff

ax = gca;

ax.FontSize = 16

>> syms S2 X2

>> S2 = (D*X_m-X_m*mu_m+S_F*Y_xs*mu_m)/Y_xs/mu_m;

>> X2 = X_m*(1-D/mu_m);

>> subplot(1,3,2)

fplot(subs(S2, [mu_m, X_m, S_F, K_S, Y_xs], [1.6, 10, 20, 1, 0.8]), [0 1.6] , 'LineWidth',2.0)

hold on

fplot(subs(X2, [mu_m, X_m, S_F, K_S, Y_xs], [1.6, 10, 20, 1, 0.8]), [0 1.6] , 'LineWidth',2.0)

fplot(subs(S2, [mu_m, X_m, S_F, K_S, Y_xs], [1.6, 10, 20, 1, 0.3]), [0 1.6] , 'LineWidth',2.0)

fplot(subs(X2, [mu_m, X_m, S_F, K_S, Y_xs], [1.6, 10, 20, 1, 0.3]), [0 1.6] , 'LineWidth',2.0)

>> xlabel('Dilution rate (1/h)')

ylabel('Cell and substrate (g/L)')

legend('Y_{xs}=0.8', 'Y_{xs}=0.8', 'Y_{xs}=0.3', 'Y_{xs}=0.3')

title('(b)');

ylim([0 16])

legend boxoff

ax = gca;

ax.FontSize = 16

>> syms S3 X3

>> S3 = (sqrt(((D-mu_m)*X_m+S_F*Y_xs*mu_m)^2+4*K_S*D*X_m*Y_xs*mu_m)-X_m*mu_m+D*X_m+S_F*Y_xs*mu_m)/(2*Y_xs*mu_m);

>> X3 = -(sqrt(((D-mu_m)*X_m+S_F*Y_xs*mu_m)^2+4*K_S*D*X_m*Y_xs*mu_m)-X_m*mu_m+D*X_m-S_F*Y_xs*mu_m)/(2*mu_m);

>> subplot(1,3,3)

fplot(subs(S3, [mu_m, X_m, S_F, K_S, Y_xs], [1.6, 10, 20, 1, 0.8]), [0 1.6] , 'LineWidth',2.0)

hold on

fplot(subs(X3, [mu_m, X_m, S_F, K_S, Y_xs], [1.6, 10, 20, 1, 0.8]), [0 1.6] , 'LineWidth',2.0)

fplot(subs(S3, [mu_m, X_m, S_F, K_S, Y_xs], [1.6, 10, 20, 1, 0.3]), [0 1.6] , 'LineWidth',2.0)

fplot(subs(X3, [mu_m, X_m, S_F, K_S, Y_xs], [1.6, 10, 20, 1, 0.3]), [0 1.6] , 'LineWidth',2.0)

>> xlabel('Dilution rate (1/h)')

ylabel('Cell and substrate (g/L)')

legend('Y_{xs}=0.8', 'Y_{xs}=0.8', 'Y_{xs}=0.3', 'Y_{xs}=0.3')

title('(c)');

ylim([0 16])

legend boxoff

ax = gca;

ax.FontSize = 16
